# Tailored Versus Generic Knowledge Brokering to Integrate Mood Management Into Smoking Cessation Interventions in Primary Care Settings: Protocol for a Cluster Randomized Controlled Trial

**DOI:** 10.2196/resprot.9715

**Published:** 2018-04-27

**Authors:** Nadia Minian, Aliya Noormohamed, Dolly Baliunas, Laurie Zawertailo, Carol Mulder, Arun Ravindran, Claire de Oliveira, Peter Selby

**Affiliations:** ^1^ Centre for Addiction and Mental Health Toronto, ON Canada; ^2^ Dalla Lana School of Public Health University of Toronto Toronto, ON Canada; ^3^ Department of Pharmacology and Toxicology University of Toronto Toronto, ON Canada; ^4^ The Association of Family Health Teams of Ontario Toronto, ON Canada; ^5^ Department of Psychiatry University of Toronto Toronto, ON Canada; ^6^ Institute for Clinical Evaluative Sciences Toronto, ON Canada; ^7^ Institute of Health Policy, Management and Evaluation University of Toronto Toronto, ON Canada; ^8^ Department of Family and Community Medicine University of Toronto Toronto, ON Canada

**Keywords:** tobacco, depression, health care practitioner, primary health care, knowledge broker, clinical decision support system, screening, brief intervention, integrated care pathways

## Abstract

**Background:**

Both tobacco smoking and depression are major public health problems associated with high morbidity and mortality. In addition, individuals with depression are almost twice as likely to smoke and less likely to achieve smoking cessation. In the Smoking Treatment for Ontario Patients program, an established smoking cessation program in Ontario, Canada, 38% of smokers in primary care settings have current or past depression with 6-month quit rates that are significantly lower than those without depression (33% versus 40%, *P*<.001). Integrating self-help mood management (eg, relaxation exercises and mood monitoring) with smoking cessation treatment increases long-term quit rates by 12%-20%. However, integration in real-world settings has not been reported. It is unclear which knowledge translation strategy would be more effective for motivating clinicians to provide resources on mood management to eligible patients.

**Objective:**

The objectives of this study are to investigate the following comparisons among depressed smokers enrolled in a smoking cessation program: 1) the effectiveness of generalized, exclusively email-based prompts versus a personalized knowledge broker in implementing mood management interventions; 2) the effectiveness of the two knowledge translation strategies on smoking quit rates; and 3) the incremental costs of the two knowledge translation strategies on the implementation of mood management interventions.

**Methods:**

The study design is a cluster randomized controlled trial of Family Health Teams participating in the Smoking Treatment for Ontario Patients program. Family Health Teams will be randomly allocated 1:1 to receive either generalized messages (related to depression and smoking) exclusively via email (group A) or be assigned a knowledge broker who provides personalized support through phone- and email-based check-ins (group B). The primary outcome, measured at the site level, is the proportion of eligible baseline visits that result in the provision of the mood management intervention to eligible patients.

**Results:**

Recruitment for the primary outcome of this study will be completed in 2018/2019. Results will be reported in 2019/2020.

**Conclusions:**

This study will address the knowledge gap in the implementation strategies (ie, email-based prompts versus a knowledge broker) of mood management interventions for smokers with depression in primary care settings.

**Trial Registration:**

ClinicalTrials.gov NCT03130998; https://clinicaltrials.gov/ct2/show/NCT03130998 (Archived on WebCite at www.webcitation.org/6ylyS6RTe)

## Introduction

### Background

Both tobacco smoking and depression are major public health problems with high morbidity and mortality [1-4]. Individuals with depression are almost twice as likely to be smokers [5-7], have lower long-term smoking abstinence (odds ratio [OR] 0.81, 95% CI 0.67-0.97) [1], and experience greater addiction severity and negative mood when quitting smoking [8-11].

Self-help mood management (eg, relaxation exercises and mood monitoring) integrated with smoking cessation treatment increases long-term quit rates by 12%-20% [3,4,12-15]. However, it remains unclear what knowledge translation (KT) strategy would be most effective in engaging practitioners to implement a mood management integrated care pathway (ICP) into primary care settings [16-20]. Two strategies that are commonly used in Canada to promote evidence-based practices include email communications and knowledge brokers (KBs) [17]. Emails provide targeted messages that connect relevant research evidence to specific practitioners, while KBs work one-on-one with practitioners to facilitate the implementation of an evidence-informed intervention [21] Several studies have shown that context where the intervention is being implemented is essential to take into account, in deciding which KT strategy to use [16,17]. Unfortunately, it is not known which of these KT strategies would be the most effective among Family Health Teams (FHTs) in Ontario, Canada. In Ontario, FHTs are primary health care organizations that include a team of family physicians, nurse practitioners, registered nurses, social workers, dietitians, and other professionals who work together to provide primary health care for their community [21].

### Objective

This study aims to assess whether email updates versus a KB (who will communicate with health care providers on an as-needed basis) is more effective at enabling practitioners within FHTs to provide their patients with mood management resources when needed. In addition, it will explore which of these KT strategies has the greatest effect on smoking abstinence and depressive symptoms at time of follow-up. We will also examine the incremental cost effectiveness of the two KT strategies, the proportion of eligible smokers who report using the resources, smoking cessation outcomes compared to patients without depressive symptoms, and practitioner improvement in knowledge, attitudes, skills, and satisfaction in addressing depressive symptoms in smokers.

In this paper, we describe the protocol for a cluster randomized trial. This design was chosen because the intervention cannot be delivered to individual practitioners within a clinic without substantial risk of contamination across study arms. The trial will be operationalized through the Smoking Treatment for Ontario Patients (STOP) program, an established smoking cessation program in Ontario, Canada. The STOP program offers up to 26 weeks of smoking cessation treatment, consisting of nicotine replacement therapy and behavioral counseling, at no cost to the patient.

## Methods

### Inclusion Criteria

#### Site Level

Family Health Teams (FHTs) in Ontario, Canada implementing an existing smoking cessation program (ie, the STOP program) and using the STOP’s online portal at the time of the study are eligible to participate. All FHTs operational in the STOP program as of November 2017 (n=153) will be eligible for randomization, except for those that do not use the online program portal at the time of patient enrollment (n=25). We anticipate that 128 FHTs will be randomized into the trial.

#### Patient Level

In order for a participant to be eligible for the trial, their baseline enrollment survey must be administered by the health care provider, in English, into the online portal in real time, so that the clinical interaction can be supported by the STOP portal. Therefore, those patients who are administered the baseline survey on paper, or in French, will be excluded. Patients, at the time of enrollment, must have depression (determined by a Patient Health Questionnaire [PHQ-9] score>9) or report a past diagnosis of depression.

### Pre-Implementation

To understand the needs of the FHTs and the importance of treating depression in the smoking cessation program, we developed a survey which measures organizational readiness and the extent to which an organization is willing and able to implement a specific intervention [22]. The survey consists of 12 questions addressing the six components of The National Implementation Research Network’s Hexagon Tool (ie, need, fit, resources, evidence, readiness, and capacity) [23], as well as the three readiness components described by Scaccia et al [24] (ie, motivation, general capacities, and specific capacities) [24]. Based on answers to this survey, organizations were grouped into two categories: most ready (n=44), and least ready (n=40). Organizations that did not answer the questionnaire (n=41) will be grouped together in a group labeled “unknown readiness.” The detailed answers from this survey will be useful for developing a KT strategy that will allow the project team to make informed decisions about their approach to change management.

In order to increase practitioners’ competencies to deliver a brief mood management, two interactive webinars will be presented to communicate best practices for integrating evidence-based mood interventions into smoking cessation programming. The webinar audience will include the STOP Community of Practice (n=300) consisting of implementers, physicians, and executive directors, who interact through bi-weekly teleconferences to communicate updates, clarify procedures, address barriers or gaps in program delivery, and share experiences with other practitioners.

### Trial Design

Study clusters (ie, FHTs) will receive either generalized messages (related to depression and smoking) exclusively through email (group A) or be assigned a KB who will provide personalized support through phone- and email-based check-ins (group B).

Practitioners from group A will receive one email per month for one year. The first email will provide an electronic copy of a relevant Cochrane review [15]*,* and a short description of the new depression ICP that will be integrated into the STOP portal. Subsequent communications will be based on general needs identified throughout the study. Practitioners from group B will receive individualized support from a KB communicating through interactive technology (ie, Skype) on an as-needed basis. The KB will be certified in tobacco cessation counseling and will have completed a specialty course on tobacco addiction treatment for individuals with mental illness [25,26].

Clinics are the unit of randomization. Two stratification factors are defined: (1) organizational readiness (described previously) with three levels, and (2) clinic size with two levels, resulting in six strata. Expected clinic size will be estimated based on past STOP enrollment because the actual number of eligible trial participants clinics will enroll is not directly observable a priori. Within each readiness stratum, the two levels of clinic size were set such that expected total enrollment in the two levels would be balanced. Within each of the six combined strata, clinics were randomly allocated in a 1:1 allocation ratio to control (group A) or intervention (group B). Treatment allocation (randomization) for each clinic was determined for all operational clinics en masse. Any clinic that began implementing the STOP program after the randomization cut-off date (Nov 14, 2017) will not be eligible for participation in the trial. The random assignment of treatment to clinic was computer generated using the ralloc command of statistical computer software Stata V.14.

### Blinding

This pragmatic, cluster trial is designed to evaluate an intervention to change practitioners’ behavior. Blinding of the clinic through its practitioners will therefore not be possible as the practitioner will be aware of the presence or absence of the KB. Participating clinics will not be informed of their treatment allocation until the trial begins. Data analysis will be blinded to treatment allocation.

### Interventions

#### Patient Screening and Brief Intervention

**Figure 1:**
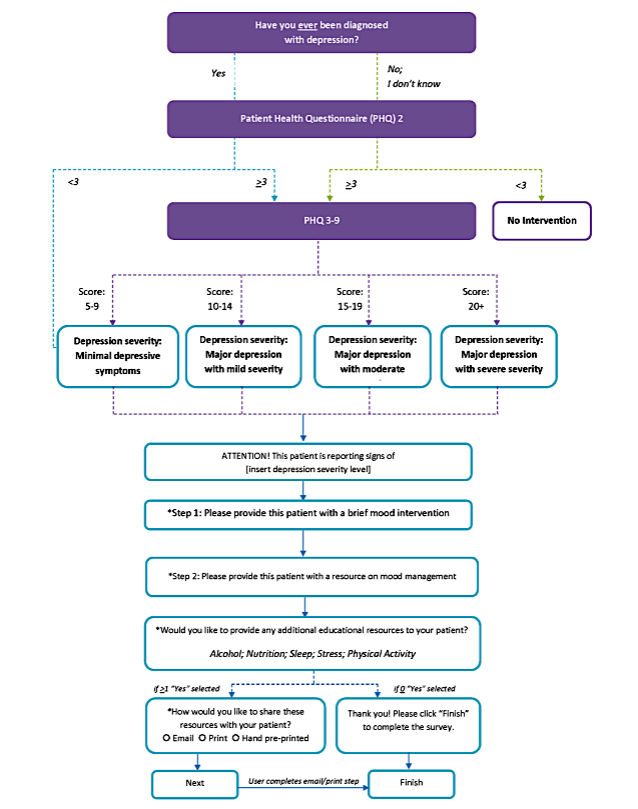
Study workflow diagram.

Across all sites, practitioners will receive the same depression ICP integrated into the STOP portal. Currently, patients are asked to self-report on past diagnosis of depression and screened for current depression using the PHQ-9. The PHQ-9 is a self-completed, 9-item instrument with each item aligning with the Diagnostic and Statistical Manual of Mental Disorders (version IV)’s criteria for depression, and developed specifically for use in primary care [27,28]. As part of the new ICP, the STOP portal will identify patients screened as having current or past depression, and prompt practitioners to provide a brief intervention and refer an evidence-based package of resources [29,30] (see Figure 1). Brief intervention messaging will be designed using the Canadian Network for Mood and Anxiety Treatments guidelines [31] and tailored to patients’ depression levels based on their PHQ-9 score. Levels can range anywhere from low risk of depression, which would warrant the usual standard of care, to major depression with severe consequences [31]. If patients’ screening results indicate risk of moderate or severe depression, practitioners will be prompted to consult with a team physician to determine next steps (eg, medication adjustment or psychiatric referral). For patients who endorse suicidal ideation, the ICP will guide practitioners to conduct an additional assessment. Whenever an intervention is warranted, practitioners will be encouraged to discuss other health risk behaviors that influence patient mood, including alcohol use and stress. Figure 1 presents a visual depiction of the study work flow.

The evidence-based resource package, which practitioners will be able to print or email it to patients, will include:

- Relaxation and mindfulness exercises
- Self-monitoring sheets to record each cigarette smoked, the activities they engaged in, and overall mood at the end of each day for 2 weeks
- Problem-solving attitudes and skills-building activities

### Outcomes

The primary outcome will be the provision of the mood management intervention to eligible patients upon completion of the STOP smoking cessation program enrollment. This dichotomous outcome will be measured as positive by a response of “Patient accepted the resource” to the practitioner question “Did the patient accept or decline the resource?”. In contrast, the outcome will be measured as negative if given a response of “Patient declined the resource” to the practitioner question “Did the patient accept or decline the resource?” or a response of “no” to the practitioner directive “Provide this patient with resources on mood management.”

The secondary outcome will be patient smoking abstinence at the 6-month follow-up survey, as measured by a negative response to the seven-day point prevalence question “Have you had a cigarette, even a puff, in the last 7 days?”. Self-reporting has been verified as a valid estimate of smoking status [32].

The tertiary outcome will be a cost-effectiveness analysis (CEA), evaluating the delivery of each intervention from the health care system, and societal perspectives. The CEA will include the costs of developing, maintaining, and running each intervention in addition to costs associated with personnel, training, supplies, and services. The incremental cost-effectiveness ratio (ICER) will be the primary outcome of the CEA. An additional measure of interest will be the 95% confidence interval for the ICER, which will be estimated using nonparametric bootstrap resampling techniques [33-36]. This method is commonly used when undertaking economic evaluations alongside clinical trials [37,38].

Other outcomes measured in this study will include change in PHQ-9 score between the baseline and 6-month follow-up surveys, the proportion of eligible smokers who report using the materials, smoking cessation outcomes compared to patients without depression, and practitioner improvement in knowledge, attitudes, skills, and satisfaction in addressing depression in smokers.

### Covariates

Patient characteristics known to affect quit outcomes include age, gender, socioeconomic status, having a quit date, alcohol and other substance use, other mental health diagnosis and the Heaviness of Smoking Index [39]. Site and patient level covariates will be treated as potential confounders in the statistical analyses.

### Sample Size

A sample size of 1224 patients per group (2446 total) was estimated using a method that accounts for intracluster correlation (ICC) within each FHT and uneven cluster sizes [40]. Using past STOP enrollment as a data source, we estimated an ICC of ρ=.032, cluster size variation coefficient of 1.24, an average annual enrolment of 24 patients, the proportion of control group patients who are provided the mood management intervention (p1) to be 0.08, and set alpha=.05 and power=.80. The minimum desired effect size was set at a risk difference=0.06. Based on enrollment in 2016-2017, we estimate the required sample size for the primary outcome will be achieved in less than 12 months.

### Statistical Analysis

All analyses will adopt an intention-to-treat principle in which sites and patients will be analyzed in the trial arm to which they are randomized. Cluster specific methods will be used because the practices, rather than patients, will be randomized, and variance in how patients are managed, and in patient quit outcomes, will be partly explained by the practice.

#### Primary and Secondary Outcomes

The association between the KT intervention and the primary outcome (ie, delivery of mood management interventions) will be analyzed using a generalized estimating equation (GEE) fitted for logistic regression, using a population-averaged method. Stratification variables will be included as covariates. An exchangeable correlation matrix and robust standard errors will be specified. All outcomes are recoded by the STOP portal system and thus a full case analysis will be used.

The association between KT intervention and the secondary outcome (ie, smoking abstinence at 6-month follow-up) will be analyzed using a GEE as described above. All patients are invited to complete the 6-month follow-up survey, but not all do, so missing outcome data are expected. Therefore, we will conduct a single imputation of the best-case scenario (all patients not smoking) and a single imputation of the worst-case scenario (all patients smoking). If the analyses from the two case scenarios imply different conclusions, multiple imputations will be performed accounting for the clustered structure of the data. All study analyses will be performed in Stata 14 [41].

#### Tertiary Outcomes

In order to conduct a cost-effectiveness analysis, we will estimate an ICER for two outcomes: number of times resources are provided to patients (health provider side) and smoking quit rates (patient side). The ICER will be calculated as the difference in discounted mean costs between intervention groups A and B divided by the difference in the outcome, using the following formula: ICER=(C_i_–C_c_)/(E_i_–E_c_), where C_i_ is the adjusted annual costs of group B, C_c_ is the adjusted annual costs of group A, E_i_ is the effect in group B, and E_c_ is the effect in group B. One-way deterministic sensitivity analyses will be performed to evaluate the robustness of our results.

### Ethical Approval and Trial Status

The study was reviewed by the Research Ethics Board at the Centre for Addiction and Mental Health (approval number: 065-2016). The trail is registered with ClinicalTrials.gov (ID: NCT03130998). At the time of manuscript submission, the readiness survey was administered, but recruitment was not completed.

## Results

Recruitment for the primary outcome of this study will be completed in 2018/2019. Results will be reported in 2019/2020.

## Discussion

It is well known that the process of integrating research evidence into practice is slow and complex [42]. Even though there have been many strategies evaluated to improve how health care professionals care for their patients, there is still no clear answer as to which is the most effective, and cost-effective, strategy to use [16,17]. Two strategies that are commonly used to promote practice change include (1) tailored and targeted messages that connect relevant research evidence to practice users [43]; and (2) KBs who work one-on-one with decision makers to facilitate evidence-informed decision-making [16].

This clinical trial will address the knowledge gap in the implementation approach associated with the use of email-based prompts versus a KB in mood management interventions for smokers with depression in primary care settings. With at least one life saved from a tobacco-related death for every two smokers who quit [44], the potential patient-level impact will extend well beyond the study duration. Assuming both approaches are equally effective at achieving a modest probability (10%) of practice change and knowing there should be a 12%-20% improvement in the likelihood of quitting smoking due to use of a mood management intervention, we would expect 19 to 32 patients with depression to quit smoking. An additional benefit to patients will be a potential improvement in their depression scores as a result of the specialized care and resources provided by clinicians as part of this study.

In addition, the Web-based portal used by STOP overcomes the issue of compatibility across various electronic medical records in FHTs. Adding a depression intervention to this system could lead to a system-wide implementation of integrated depression care pathways at a relatively low cost, potentially reaching 2.25 million Ontarians registered at these FHTs. Moreover, a technology-based KB model will help reduce travel costs and expand the reach of KBs in the future.

Finally, rapid and efficient implementation in other settings participating in the STOP program, such as Community Health Centres, addiction agencies, Public Health Units, and Nurse Practitioner–Led Clinics, is possible at a relatively low cost. We will also have high quality data on these populations for planning and monitoring the effects of interventions in primary care settings.

Some potential limitations should be acknowledged. As noted earlier, health care practitioners will not be blinded to the treatment allocation. During this study, a health care practitioner might work in two different clinics, one assigned to group A, and one to group B. In this case, there is the possibility of contamination of knowledge, as the health care practitioner might apply knowledge obtained from the KB advice received while working in a group B clinic to patients in their group A clinic. This possible contamination could decrease the trial effect and lead to a more conservative effect estimate. However, we anticipate that this will be a rare occurrence. In addition, there is a risk that detecting change in the abstinence rates may be underpowered when estimating the ICER for the economic evaluation.

## Abbreviations

CEA: cost-effectiveness analysis
FHT: Family Health Team
GEE: generalized estimating equation
ICC: intracluster correlation
ICER: incremental cost-effectiveness ratio
ICP: integrated care pathway
KB: knowledge broker
KT: knowledge translation
OR: odds ratio
PHQ-9: Patient Health Questionnaire
STOP: Smoking Treatment for Ontario Patients
